# Retraction: Rendon-Marin et al. Canine Morbillivirus from Colombian Lineage Exhibits In Silico and In Vitro Potential to Infect Human Cells. *Pathogens* 2021, *10*, 1199

**DOI:** 10.3390/pathogens11111348

**Published:** 2022-11-14

**Authors:** Santiago Rendon-Marin, Carolina Quintero-Gil, Diego Guerra, Carlos Muskus, Julian Ruiz-Saenz

**Affiliations:** 1 Grupo de Investigación en Ciencias Animales—GRICA, Facultad de Medicina Veterinaria y Zootecnia, Universidad Cooperativa de Colombia, sede Bucaramanga, 680002 Bucaramanga, Colombia; 2 Programa para el Estudio y Control de Enfermedades Tropicales—PECET, Facultad de Medicina, Universidad de Antioquia, 050010 Medellín, Colombia

The authors retract the article “Canine Morbillivirus from Colombian Lineage Exhibits In Silico and In Vitro Potential to Infect Human Cells” [1]. Following publication, the authors contacted the editorial office regarding contaminated batches of the virus. The experiments conducted were therefore performed with a virus batch that does not correspond to the wild type of the virus, but to a reference virus adapted to the cell culture. 

This article [1] has been retracted and shall be marked accordingly. We apologize to the readers of *Pathogens*.

This retraction was approved by the Editor-in-Chief of the journal and all authors agreed to this retraction. 
